# The Emergence of Multiple Antibiotic Resistance in Culture Sensitivities of Post-surgical Patients in Lahore General Hospital, Lahore

**DOI:** 10.7759/cureus.23212

**Published:** 2022-03-16

**Authors:** Tauseef Fatima, Zain ul A Askri, M Hasaan Shahid, Anwar Z Khan, Suleman Asif, Ahsan R Ghumman, Muhammad Farooq Afzal

**Affiliations:** 1 Surgery, Lahore General Hospital, Lahore, PAK; 2 Surgical Unit 1, Lahore General Hospital, Lahore, PAK; 3 General Surgery, Lahore General Hospital, Lahore, PAK

**Keywords:** culture and sensitivity, observational study, surgical site infection, antimicrobial resistance, antibiotics

## Abstract

Objective

The purpose of this study is to isolate the organisms which are developing resistance and to recognize the drugs against which resistance has emerged so that antibiotic policy can be formulated for the proper and effective use of antibiotics.

Setting and design

An observational study was conducted for a period of six months from July 1, 2021 and December 31, 2021 in LGH.

Methods

Statistics regarding the culture and sensitivity of the organisms isolated from different sources were collected from the surgery department. 195 cultural and sensitivity reports were analyzed for identification of genus/species of bacteria and sensitivity of the organism.

Results

Out of 195 culture reports, 124 showed significant growth of organisms exhibiting resistance to either single or multiple drugs. Escherichia and acinobactor was the most common organism isolated with a total of 30 each (24%, 24%), followed by pseudomonas 21 (17%), Klebsiella was 13 (10%), Proteus was 10 (8%), Methicillin-resistance Staph-aureus was seven (5%), Methicillin-sensitive Staph-aureus was five (4%), Staphylococcus epidermidis was four (3%), Providencia, Streptococci, Enterobacter species and Citrobacter species were one (1%).

Maximum resistance was detected with frequently used first-line antimicrobials such as Ceftriaxone, ampicillin and Clavulanic acid. Least resistant were Azithromycin, Cefoxitin, Cefaclor among the gram-negative and gram-positive bacteria.

Conclusion

Antimicrobial resistance (AMR) was more against frequently used antibiotics that are accessible for an extended duration. Variation of resistance and sensitivity pattern with time is identified. Periodic AMR monitoring and rotation of antibiotics are suggested to restrict further emergence of resistance.

## Introduction

Antimicrobial resistance (AMR) is a growing public health concern where the microorganism is able to survive exposure to antibiotic treatment [1]. There are multiple reports about the emergence of antibiotic resistance from different countries including the US, Brazil, India, Jorden. These reports include vancomycin-resistant Staphylococcus aureus (VRSA) and vancomycin-resistant Enterococci [2-5]. Controlling infections is one of the toughest jobs in developing countries like Pakistan where AMR in surgical site infections (SSIs) still holds high mortality and morbidity [6]. Methicillin resistance S. aureus (MRSA) identified in 1990 started as a single clonal mutation that resulted in community-acquired MRSA. These resistant organisms result in SSI causing high mortality and morbidity [6]. SSIs are the most common hospital-acquired infections (HAI) occurring in 1.9% of all surgeries causing an increase in morbidity, reoperation, rates of readmission, utilization of resources and eventually increasing the financial burden on the health care system [7].

The skin has a major role as a physical barrier against harmful bacteria but higher temperature and humidity in a post-operative wound create a favorable environment for the growth of gram-negative bacilli, Corynebacterium spp. and S. aureus [8]. SSI has been classified into superficial, deep, or organ space by The United States Center for Disease Control. A superficial infection occurs within 30 days of surgery involving only the skin and subcutaneous tissues [9].

SSI in clean surgeries is not uncommon. Gastrointestinal surgery has a higher rate of SSI than other procedures. Among the types of gastrointestinal procedures, colorectal surgery has the highest risk due to the presence of multiple bacteria in the large gut and rectum [10]. We are now in a “post-antibiotic era” and living on the verge of facing a global “antibiotic resistance (AR) crisis.” As estimated, MRSA has been the leading pathogen responsible for the AR crisis causing 60%-90% of nosocomial infections. According to WHO, till the end of 2050, drug-resistant infections can lead to 10 million annual deaths which may result in a global financial disaster affecting millions of people [11]. Multi-drug therapy has a broad spectrum for microbial agents and can also be used as combination therapy for a specific pathogen. Drug resistance has been the most common indication for combination therapy since it lowers the overall risk of drug resistance emergence in a pathogen.

In Lahore general hospital we are facing a big challenge regarding dealing with SSI most of which are caused by organisms that are antimicrobial-resistant. Social factors such as deficient hygienic practices, demographic variations and overcapacity have been enumerated for the emergence of AMR. Irrational and inappropriate usages of antibiotics in humans and animals for treatment and other usages (as growth promoters) have been thought as the main causes for the rise of hospital and community-acquired resistant infections by the World Health Organization (WHO).

The main cause of Multidrug resistance SSIs is not known in LGH. In view of this fact, this study was conducted to identify the group of organisms that have developed the resistance in surgical unit 1 in LGH, to identify the classes of the drug against which this resistance has emerged. On the basis of these findings, we will be able to formulate the proper and effective use of antibiotics.

## Materials and methods

Study design and duration

We conducted an observational study for a period of six months from July 1, 2021 to December 31, 2021 after the approval from ethical committee.

Data collection

The data regarding culture and sensitivity of the organisms isolated from different sources such as blood, wound swab/pus, urine other sources such as sputum or tips of CVPs were collected. One hundred and ninety-five culture sensitivity reports were included from the patients.

Inclusion and exclusion criteria

These patients were admitted to Surgical Unit 1, Lahore General Hospital, Lahore, and remained admitted for period of more than seven days and acquired a surgical site wound infection. These samples were sent to laboratories for identifications of organisms and antimicrobial sensitivity. Organisms resistant to more than one drug were considered MDR. Patients that were not remained admitted to the hospital for seven days were excluded. 

Statistical analysis

Collected data were statistically analyzed by IBM SPSS Statistics ver. 24.0 (IBM Corp, Chicago, IL). Descriptive statistics were used for the analysis of results.

## Results

**Figure 1:**
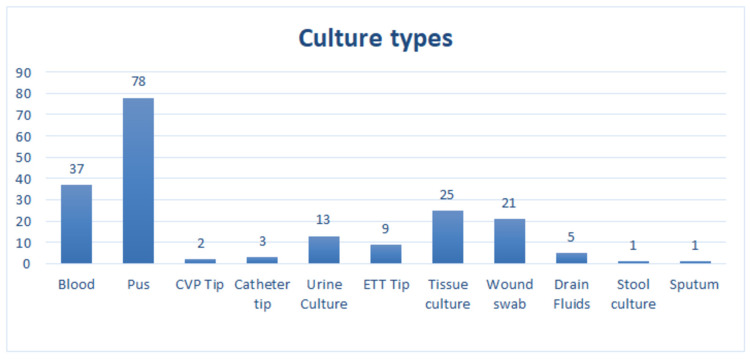
Types of samples collected from patients

Total 195 cultures and sensitivities were analyzed in 85 patients. Among them 59 (69.4%) were male and 26 (30.5%) were female. Out of 195 culture reports, 65 showed no growth while 124 showed 12 different bacteria and 6 (4%) showed the presence of Candida species (Figure 1).

**Figure 2:**
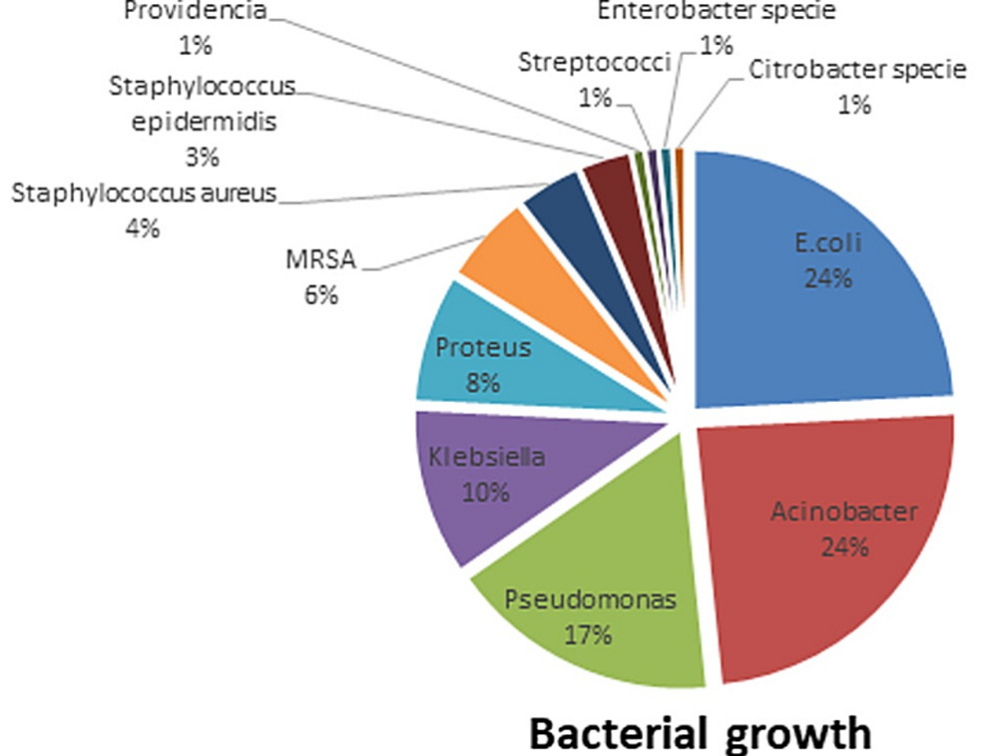
Distribution of organisms among various samples

Among 124 reports that isolated bacteria, Escherichia coli was 30 (23%), Acinetobacter species was 30 (23%). Pseudomonas was 21 (16%), Klebsiella species 13 (10%), Proteus species 10 (8%), MRSA seven (5%), S. aureus five (4%), Staphylococcus epidermidis four (3%) (Figure 2).

**Figure 3:**
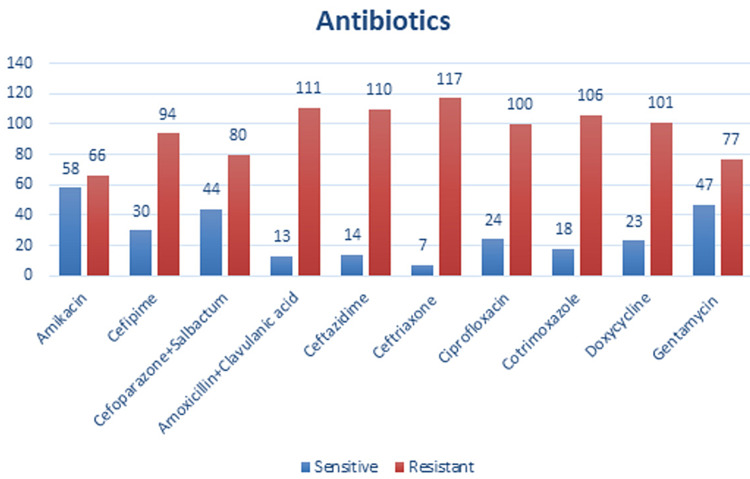
Drugs having resistance in samples

Antibiotics that were tested for 124 reports that isolated bacteria were Amikacin sensitive in 58 (46.7%) while resistant in 66 (53.22%), Cefipime sensitive in 30 (24.19%) while resistant in 94 (75.80%), Cefoparazone+Salbactum sensitive in 44 (35.48%) while resistant in 80 (64.51%), Amoxicillin+Clavulanic acid sensitive in 13 (35.48%) while resistant in 111 (89.51%), Ceftazidime sensitive in 14 (11.29%) while resistant in 110 (88.70%), Ceftriaxone sensitive in seven (0.80%) while resistant in 117 (94.35) (Figure 3).

**Figure 4:**
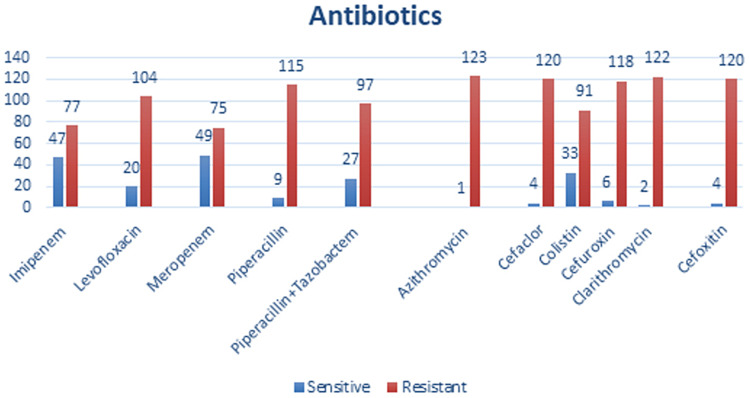
Drugs having resistance/sensitivity in collected samples

The most resistance drugs were Piperacillin 115 (92.74%), Azithromycin 123 (99.19%), Clarithromycin 122 (98.38%), Piperacillin+Tazobactem 120 (96.77%), Cefuroxin 118 (95.16%) and Cefoxitin 120 (96.77%) (Figure 4).

**Figure 5:**
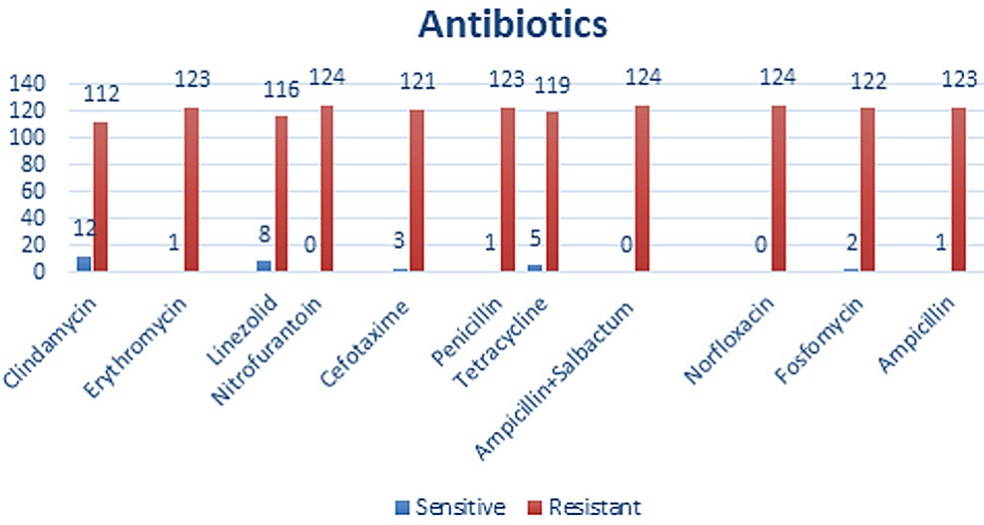
Drug having resistance/sensitivity

The most resistance drugs were Clindamycin in 112 (90.32%), Erythromycin 123 (99.19%), Linezolid 116 (99.53%), Nitrofurantoin 124 (100%), Cefotaxime 121 (97.58%), Penicillin 123 (99.19%), Tetracycline 119 (95.96%), Ampicillin+Salbactum 124 (100%), Norfloxacin 124 (100%), Fosfomycin 122 (98.38%), and Ampicillin in 123 (99.19%) (Figure 5).

Bacteria and antibiotic sensitivity

In our collected data, E. coli was sensitive to the drugs shown in Table 1 with percentages. It was sensitive to Amikacin 21 (70%) the most and Ciprofloxacin five (16.6%) the least. E. coli was resistant to all other antibiotics. Acinetobacter was sensitive to Amikacin nine (30%) only but most sensitive to Colistin 16 (53.33%), while resistant to all other antibiotics.

**Table 1:** Bacteria with their sensitivity to antibiotics

	E. coli 30 (23%)	Acinitobacter 30 (23%)	Pseudomonas 21 (16%)	Klebsiella 13 (10%)
Amikacin	21 (70%)	9(30%)	11(52.38%)	7(53.84%)
Cefipime	06 (20%)	3(10%)	8(38.09%)	3(23.07%)
Cefoparazone + Salbactum	12 (40%)	7(23.33%)	10(47.61%)	5(16.66%)
Amoxicillin + Clavulanic acid	02 (6%)	(R)	(R)	2(15.38%)
Ceftazidime	1 (3%)	2(6.6%)	4(19.04%)	2(15.38%)
Ceftriaxone	2 (6%)	2(6.6%)	(R)	2(15.38%)
Ciprofloxacin	5 (16.6%)	2(6.6%)	10(47.61%)	2(15.38%)
Cotrimoxazole	(R)	2(6.6%)	1(4.7%)	2(15.38%)
Doxycycline	(R)	8(26.6%)	1(4.7%)	2(15.38%)
Gentamycin	(R)	6(20%)	12(57.14%)	4(30.76%)
Imipenem	(R)	8(26.6%)	11(52.38%)	6(46.15%)
Levofloxacin	(R)	2(6.6%)	8(38.08%)	2(15.38%)
Meropenim	(R)	6(20%)	12(57.14%)	4(30.76%)
Piperacilin	(R)	3(10%)	6(28.57%)	
Piperacilin + Tazobactum	(R)	5(16.6%)	8(38.09%)	2(15.38%)
Colistin	(R)	16(53.33%)	9(42.85%)	3(23%)
Cefuroxime	(R)	(R)	(R)	1(7.6%)
Cefotaxime	(R)	(R)	(R)	1(7.6%)
Tetracycline	(R)	(R)	(R)	1(7.6%)
Fosfomycin	(R)	(R)	(R)	1(7.6%)

Pseudomonas was sensitive to Meropenem 12 (57.14%), Gentamycin 12 (57.14%), Imipenem 11 (52.38%) and Amikacin 11 (52.38%) the most and to Cotrimoxazole one (4.7%), Doxycycline one (4.7%) the least, while resistant to all other antibiotics. Klebsiella was sensitive to Amikacin seven (53.84%) the most and to Cefuroxime one (7.6%), Cefotaxime one (7.6%), Tetracycline one (7.6%), Fosfomycin one (7.6%) the least, while resistant to all other antibiotics (Table 1).

Proteus species was sensitive to Cefoparazone+Salbactum nine (90%), Amikacin eight (80%), Cefipime seven (70%) the most and to Doxycycline one (10%) the least, while resistant to all other antibiotics. MRSA was sensitive to Doxycycline four (57.14), Clindamycin four (57.14%) the most and Tetracycline one (14.28%) the least, while resistant to all other antibiotics.

Staphylococcus aureus was sensitive Clindamycin five (100%) the most, Azithromycin one (20%) the least. Staphylococcus epidermidis was sensitive to almost all the drugs (Table 2).

**Table 2:** Bacteria with their sensitivity to antibiotics

	Proteus 10 (8%)	MRSA 7 (5%)	Staphylococcus aureus 5 (4%)	Staphylococcus epidermidis 4 (3%)
Amikacin	8(80%)	(R)	(R)	(R)
Cefipime	7(70%)	(R)	(R)	(R)
Cefoparazone + Salbactum	9(90%)	(R)	(R)	(R)
Amoxicillin + Clavulanic acid	4(40%)	(R)	1(20%)	3 (75%)
Ceftazidime	4(40%)	(R)	(R)	(R)
Ciprofloxacin	2(20%)	(R)	(R)	1(25%)
Cotrimoxazole	(R)	2(28.5%)	1(20%)	3(75%)
Doxycycline	1(10%)	4(57.14%)	(R)	3(75%)
Gentamycin	4(40%)	2(28.5%)	3(60%)	4(100%)
Azithromycin	(R)	(R)	1(20%)	(R)
Cefaclor	(R)	(R)	1(20%)	3(75%)
Cefoxitin	(R)	(R)	1(20%)	3(75%)
Imipenem	6(60%)	(R)	(R)	(R)
Levofloxacin	2(20%)	(R)	(R)	2(50%)
Meropenim	7(70%)	(R)	(R)	(R)
Piperacilin + Tazobactum	3(30%)	(R)	(R)	(R)
Colistin	(R)	1(14.28%)	(R)	(R)
Clindamycin	(R)	4(57.14%)	5(100%)	3(75%)
Erythromycin	(R)	1(14.28%)	(R)	(R)
Linezolid	(R)	2(28.5%)	3(60%)	3(75)
Cefuroxime	(R)	(R)	1(20%)	3(75%)
Cefotaxin	(R)	(R)	1(20%)	(R)
Clarithromycin	(R)	(R)	1(20%)	1(25%)
Tetracycline	(R)	1(14.28%)	(R)	(R)

Providencia species was sensitive to Cefipime one (100%), Amoxicillin+Clavulanic acid one (100%), Ceftazidime one (100%), Ceftriaxone one (100%), Ciprofloxacin one (100%), Cotrimoxazole one (100%), Gentamycin one (100%), Imipenem one (100%), Meropenim one (100%), Cefotaxime one (100%) while resistant to all other antibiotics.

Streptococci were only sensitive to Cefipime one (100%), while resistant to all other antibiotics. Enterobacter was sensitive to Amikacin one (100%), Gentamycin one (100%), Meropenim one (100%) while resistant to all other antibiotics.

**Figure 6:**
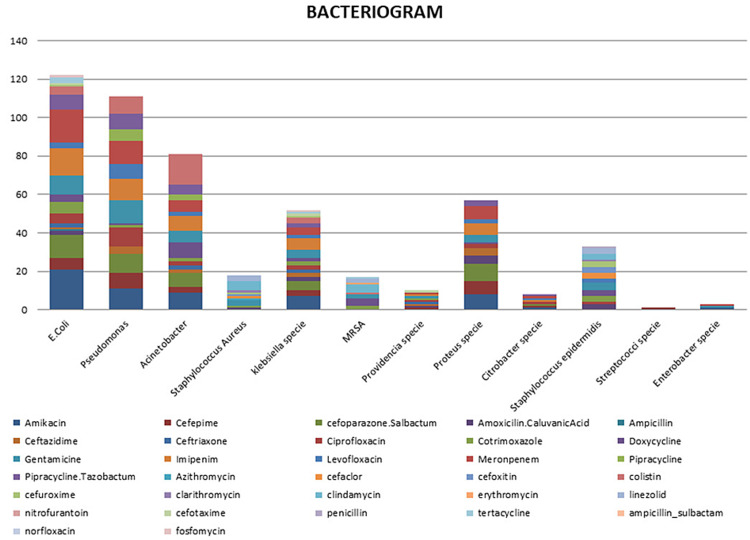
Bacteriogram showing resistance against drugs

Citrobacter was sensitive to Amikacin one (100%), Cefipime one (100%), Cefoparazone+Salbactum one (100%), Ciprofloxacin one (100%), Imipenem one (100%), Levofloxacin one (100%), Meropenim one (100%), Piperacillin+Tazobactum one (100%), while resistant to all other antibiotics (Table 3, Figure 6).

**Table 3:** Bacteria with their sensitivity to antibiotics

	Providencia species 1(1%)	Streptococci 1 (1%)	Enterobacter 1 (1%)	Citrobacter 1 (1%)
Amikacin	(R)	(R)	1(100%)	1(100%)
Cefipime	1(100%)	1(100%)	(R)	1(100%)
Cefoparazone + Salbactum	(R)	(R)	(R)	1(100%)
Amoxicillin + Clavulanic acid	1(100%)	(R)	(R)	(R)
Ceftazidime	1(100%)	(R)	(R)	(R)
Ceftriaxone	1(100%)	(R)	(R)	(R)
Ciprofloxacin	1(100%)	(R)	(R)	1(100%)
Cotrimoxazole	1(100%)	(R)	(R)	(R)
Gentamycin	1(100%)	(R)	1(100%)	(R)
Imipenem	1(100%)	(R)	(R)	1(100%)
Levofloxacin	(R)	(R)	(R)	1(100%)
Meropenim	1(100%)	(R)	1(100%)	1(100%)
Piperacilin + Tazobactum	(R)	(R)	(R)	1(100%)
Cefotaxime	1(100%)	(R)	(R)	(R)

 

## Discussion

The diagnosis of infection in critical patients is important because early appropriate antimicrobial therapy improves outcomes. The accurate and timely provided pathological data by the laboratory supports better outcomes for post-surgical patients, especially in an era of increased AR.

In our study, the main prevalence of SSI in patients was gram-negative bacteria, i.e., 84% while gram-positive bacteria were 16%. In studies done in India [12] and Nigeria [13] the prevalence of gram-negative bacteria was 24% and 65%, respectively. This represents that in our healthcare setups, gram-negative bacteria are emerging multi-drug resistant bacteria. E. coli and Acinetobacter were the most commonly acquired bacteria causing surgical site infection in our study, i.e., E. coli 24% and Acinetobacter 24%. In another study conducted in Nigeria [14], the prevalence of E. coli was 40.35%. Recent studies in Iran [15] and Ethiopia [16,17] showed a prevalence of Acinetobacter 75.7% and 3.8%, respectively, showing the difference of prevalence between African and Asian countries. A study in Nigeria showed Imipenem was the most active antibiotic against E. coli isolates as 71% of them were susceptible to this antibiotic [14] while in our setup E. coli was most sensitive to Amikacin 70% and Cefoparazone+Salbactum 40% but resistant to Imipenem.

According to an Iranian study, Acinetobacter was most sensitive to Amikacin 6% with resistance incidence of 88.9% and Ceftazidime 3.4% and Cephalexin 3.4% with resistance incidence of 97% in each [15] while study results in Ethiopia showed Acinetobacter was least resistant against Meropenem 33.3% and Ciprofloxacin 44.5% and 100% resistant to Ampicillin and Piperacillin [16].

In our study, Colistin 53.33% Amikacin 30%, Imipenem 26.6% and Doxycyclin 26.6% were most active against Acinetobactor with only 10% sensitivity against Piperacillin and 100% resistance against Ampicillin.

Pseudomonas aeruginosa was found in 17% of culture sensitivity reports in our study while recent studies in Iran [17] and Ethiopia [16] showed 24.3% and 6% prevalence of Pseudomonas respectively. According to a study in Iran Pseudomonas was sensitive to Amikacin 48% and Gentamycin 46.7% with a resistance incidence of 52% in each drug and in Ethiopia, it was least resistant against Ciprofloxacin 36.4% and Meropenem 45.5% [16,17]. In our data, it was most sensitive to Gentamycin 57.14%, Amikacin 52.38%, Cefoparazone+Salbactum 47.61% and Ciprofloxacin 47.61% showing relatively similar results as in Iran.

Klebsiella was found in 8% culture sensitivity reports of our study while a study done in Egypt [18] the prevalence of Klebsiella was 24% and in Nigeria [19] it was 17.3%. According to a study in Egypt, Klebsiella species was 83.3% sensitive to Colistin Sulphate, 66.7% to Levofloxacin, 58.3% to Ciprofloxacin and 50% sensitive against Amikacin [18]. In our study, Klebsiella was most sensitive to Amikacin 53.84%, Imipenem 46.15%, Meropenem 30.76% while only 23% was sensitive to Colistin.

Proteus was found in 8% of culture sensitivity reports of our study while according to a study in Nigeria [19] the prevalence of Proteus was 7.7% in SSI culture sensitivity reports. In our study, Proteus was most sensitive to Cefoparazone+Salbactum 90% and Amikacin 80%.

Providencia was found in 1% culture sensitivity reports of our study while data from a research study in Egypt [18] showed Providencia had a 14% prevalence in SSI culture sensitivity reports. In Egypt, Providencia was most sensitive to Colistin and Amikacin [18] while in our study Providencia was 100% sensitive to Cefepime, Amoxicillin Clavulanic acid, Ceftazidime, Ceftriaxone, Ciprofloxacin, Cotrimoxazole, Gentamycin, Imipenem and Meropenim. S. aureus contributed for 10% of SSI among them, 6% were MRSA while 4% were MSSA. A study done in Asmara, Eritrea showed the prevalence of Staph infection was 63.1% and out of all Staph positive cultures 72% were MRSA [20] and according to Indian research staph infection in surgical wounds was 22.90% [21], which shows that Staph infection is not as much prevalent in our hospitals as compared to African healthcare centers. In Africa, S. aureus sensitivity to Methicillin was 19.5% and 15.9% were against Vancomycin [16]. In our study, S. aureus was most sensitive to Clindamycin 100% and Gentamycin 60% while MRSA was most sensitive to Doxycycline 57.14% and Clindamycin 57.14%.

This study will not only help in the determination of antibiotic prescription in the surgical unit, but it also helps in the promotion of rational use of antibiotics in the community and all personnel who are authorized to prescribe the antibiotics. This will help the administration in formulating the appropriate antibiotics policy. There are a few limitations of these studies including the sample size which is small as compared to other studies. We were not able to correlate the resistance to prior antibiotic exposure of these patients.

## Conclusions

Hospital-acquired SSIs are a major risk factor for the negative outcomes in post-surgical patients. Every member of the surgical team has a responsibility to understand the importance of evidence-based practices during preoperative, intraoperative, and postoperative phases. It includes devising an antibiotic prophylaxis policy. Strict implementation of operating room regulations is required especially in emergency settings, such as hand hygiene, operating room sterilization, restriction in the number of personnel during procedures and taking proper measures regarding pre-operative patient skin antisepsis.

Perioperative minimum use of drains is also recommended to prevent SSI in patients. Cultures and swabs should be taken before starting antibiotics and labeled accurately. Prolong use of antibiotics has a major role in causing the emergence of resistant organisms in post-operative patients. In present-day surgical practice, the implementation of standard guidelines in our hospitals is required to lower the risk of antibiotic resistance crisis.
